# Purchasing for high-quality care using National Health Insurance: evidence from Zambia

**DOI:** 10.1093/heapol/czad022

**Published:** 2023-04-06

**Authors:** Doris Osei Afriyie, Felix Masiye, Fabrizio Tediosi, Günther Fink

**Affiliations:** Department of Epidemiology and Public Health, Swiss Tropical and Public Health Institute, Kreuzstrasse 2, Allschwil 4123, Switzerland; University of Basel, Petersplatz 1, Basel 4001, Switzerland; Department of Economics, University of Zambia, Lusaka, Zambia; Department of Epidemiology and Public Health, Swiss Tropical and Public Health Institute, Kreuzstrasse 2, Allschwil 4123, Switzerland; University of Basel, Petersplatz 1, Basel 4001, Switzerland; Department of Epidemiology and Public Health, Swiss Tropical and Public Health Institute, Kreuzstrasse 2, Allschwil 4123, Switzerland; University of Basel, Petersplatz 1, Basel 4001, Switzerland

**Keywords:** Health insurance, quality of care, purchasing, health financing, Zambia

## Abstract

Improving the quality of care is essential for progress towards universal health coverage. Health financing arrangements offer opportunities for governments to incentivize and reward improvements in the quality of care provided. This study examines the extent to which the purchasing arrangements established within Zambia’s new National Health Insurance can improve equitable access to high-quality care. We adopt the Strategic Purchasing Progress and the Lancet Commission for High-Quality Health Systems frameworks to critically examine the broader health system and the purchasing dimensions of this insurance scheme and its implications for quality care. We reviewed policy documents and conducted 31 key-informant interviews with stakeholders at national, subnational and health facility levels. We find that the new health insurance could boost financial resources in higher levels of care, improve access to high-cost interventions, improve care experiences for its beneficiaries and integrate the public and private sectors. Our findings also suggest that health insurance will likely improve some aspects of structural quality but may not be able to influence process and outcome measures of quality. It is also not clear if health insurance will improve the efficiency of service delivery and whether the benefits realized will be distributed equitably. These potential limitations are attributable to the existing governance and financial challenges, low investments in primary care and shortcomings in the design and implementation of the purchasing arrangements of health insurance. Although Zambia has made progress in a short span, there is a need to improve its provider payment mechanisms, and monitoring and accounting for a higher quality of care.

Key messages

- The health insurance scheme could potentially improve access to high-cost interventions and integrate the public and private sectors.
- More progress towards strategic purchasing for quality of care by Zambia’s National Health Insurance is likely possible with government contribution to the scheme for vulnerable groups, increased investments in primary health care and strong governance for quality.
- Health insurance can positively influence the quality of care through a balance of structural, process and outcome indicators to monitor providers and the use of the claims data across its mix of providers.

## Introduction

Poor quality of care continues to be a primary cause of high mortality in low- and middle-income countries (LMICs), with an estimated 8.6 million excess deaths contributed to low quality of care in 2016 (Kruk *et al.*, 2018b). This high excess mortality highlights the persistent gaps between the effective coverage of essential interventions and the low fraction of potential health gain that is currently delivered to populations (Ng *et al.*, 2014).

At the macro-system level, health financing, and purchasing in particular, is one of the main strategies that can be used to influence greater quality in the health system (Lagomarsino *et al.*, 2012; Kutzin, 2013; Mbau *et al.*, 2018). When countries establish mechanisms for resource pooling, it places them in a better position to strategically purchase quality services. Purchasing is considered ‘strategic’ if the allocation of funds to health service providers by purchasers is linked to provider performance or population needs (Mathauer *et al.*, 2019). Purchasers can be institutions such as Ministries of Health (MOHs), mandatory health insurance agencies or other autonomous insurance agencies. As health financing reforms are implemented within the broader health system context, which is dynamic and complex in nature, it is important to examine how this context shapes reforms and their ability to achieve their goals (Duran *et al.*, 2020).

Health insurance schemes have been introduced in many LMICs in recent years, offering new opportunities for governments to become ‘strategic purchasers’ and to improve access to high-quality care to make progress towards universal health coverage (UHC) (Mathauer *et al.*, 2019). Zambia is one of the countries that has recently introduced health insurance. In 2018, Zambia passed its National Health Insurance (NHI) Act with the aim of providing ‘universal access to quality health services’ (Government of Zambia, 2018). The Act established the National Health Insurance Management Authority (NHIMA), a semi-autonomous agency, which is now in charge of collecting contributions from residents, purchasing services from various health institutions and providing entitlements to beneficiaries. According to the current statutory instrument, employees are mandated to contribute 1% of their monthly salary with employers equally matching (Government of Zambia, 2019). Those who are self-employed and in the informal sector must contribute 1% of their declared monthly income, with 60 kwacha (USD 4) being the minimum contribution. Deductions from salaries in the formal sector began in October 2019, and the disbursement of funds to health facilities commenced in February 2020. Principal members can have six beneficiaries under their membership, and as of February 2022, the number of registered principal members and secondary registered beneficiaries was 1.35 million and 500 000, respectively (National Health Insurance Management Authority, 2022). Individuals >65 years and those who are mentally ill and physically disabled are exempted from contributions.

As health insurance is in its early phase of implementation and considers reforms that facilitate its goal of steering towards UHC, it is critical to identify the implications of its purchasing functions and assess its future impact on high-quality care for all. In this article, we examine the design of the purchasing arrangements within Zambia’s NHI and its implications for accessing high-quality care.

## Methods

### Study design and setting

Zambia is a lower-middle-income country in Southern Africa with a population of 18 million, of which over half live in rural areas. Table 1 shows the key indicators for Zambia.

**Table 1.** Key indicators for Zambia (International Monetary Fund, 2020; The World Bank, 2020)

		Year
**Macro-fiscal indicators**		
GDP per capita (current USD)	985	2020
Total public debt (% GDP)	95.5	2020
Poverty rate at USD 1.90 per day	58.7	2015
**Demography**		
Population (millions)	18	2020
Urban population (% of total population)	45	2020
**Health financing indicators**		
Current health expenditure, as percentage of GDP	4.5	2016
Government health expenditure, as percentage of current health expenditure	41	2016
Out-of-pocket health expenditure, as percentage of total health expenditure	12	2016
Key health indicators		
Life expectancy at birth, total (years)	64	2019
Maternal mortality ratio (per 100 000 live births)	213	2017
Neonatal mortality rate (per 1000 live births)	24	2020
Births attended by skilled health workers as percentage of total births	80.4	2018

The main health service providers are public although there are many faith-based mission and private providers. The public health system is organized as a pyramid structure with three main levels. The bottom level constitutes the primary care that includes the first-level/Level 1 hospitals, health centres and health posts. In 2012, user fees were abolished in all public primary care facilities (Chitah *et al.*, 2018). Level 2 hospitals are one level above these facilities and mainly used for curative care in paediatrics, obstetrics and general surgery, followed by the tertiary level, which includes the teaching hospitals that provide specialized care such as cancer treatment, dialysis and orthopaedics. Public health institutions are financed through monthly operational grants from the Ministry of Finance (MOF) that are on a needs-based resource allocation formula.

### Study population

Key-informant interviews were carried out with 31 stakeholders at national, subnational and health facilities from November 2020 to February 2021. Key informants were purposely selected, focusing on those involved in health policy, health financing, design and implementation of the health insurance. At the national level, interviews were conducted with stakeholders from governmental [MOH, MOF and Ministry Labour and Social Security (MLSS)], private-sector, multilateral and non-governmental organizations. Three provinces were purposely selected based on the distance from the capital and performance on health outcomes such as maternal mortality and under-five mortality. In each province, stakeholders at the provincial health office and facility managers of Level 2 hospitals were interviewed. Within each province, one district, which has an accredited first-level hospital, was conveniently selected.

We also conducted a document review of published articles, policy documents and country reports. Documents were identified through the interviews with the stakeholders. Additionally, we searched PubMed and Google Scholar databases, using the search terms ‘health systems’ or ‘health financing’ or ‘health insurance’ and ‘Zambia’.

### Study conceptual framework

To examine the potential ability of Zambia’ NHI to influence equitable access to high-quality care, we adapted the strategic health purchasing (SHP) progress framework (Cashin *et al.*, 2018). The framework was developed to examine the critical functions necessary for strategic purchasing of health care by purchasing agencies such as NHI. The framework focuses on purchasing as a policy lever to improve UHC’s intermediate and ultimate objectives such as equity, efficiency and quality.

The framework consists of two main dimensions that are critical for purchasing to contribute to the quality of service delivery. The first dimension is the health system functions that support the ability of purchasing to influence the quality of services, and the second dimension is the purchasing functions.

The health system functions that are critical to support strategic purchasing are (1) governance and information, (2) service readiness and provision and (3) sufficiency and institutional flow of resources. Governance and information comprise the regulatory policies and systems needed to support quality as well as strengthening systems for establishing licensure and accreditation systems. Service readiness and provision pertain to improving processes for evidence-based care and having adequate inputs such as medicines available to enable the delivery of high-quality services. Last, the financial flows to providers ensure sufficient resources for health and a reduction in the fragmentation of pooled funds. In addition, giving providers autonomy in spending and managerial decision-making is important.

There are four main domains under the purchasing functions of the SHP framework. These purchasing functions include (1) governance of purchasing, (2) healthcare goods and services to purchase, (3) providers from whom goods and services are purchased and (4) how to purchase. Governance of purchasing includes alignment of purchasing with UHC goals, assigning clear roles and responsibilities for participating institutions and ensuring that institutions and staff have the technical capacity to fulfil their duties. For healthcare goods and services to purchase, countries have to define and create systems for revising benefit packages and the list of covered medicines by relevant stakeholders. In addition, there needs to be a description of requirements for purchasing such as adherence to standard treatment guidelines and referral guidelines including gatekeeping policies. In regard to providers to purchase from, this involves the use of quality requirements of the benefit package to determine the eligibility for service providers for each level of care and the decision to include private providers. Last, the design of how to purchase services and goods encompasses the basis of payment, which includes payment rates, and how to hold providers accountable for service quality.

To conceptualize quality of care, we adapted the Lancet Commission for High-Quality Health Systems (HQSS) framework (Kruk *et al.*, 2018a). The framework asserts that quality improvement requires system-level interventions involving leadership at all levels of the health system and interventions that value people. We focus on the processes of the care domain and the framework proposes for it to be asserted along with two main components: competent care and systems and positive user experiences. Competent care and systems require evidence-based and effective care that includes correct diagnosis, appropriate treatment and counselling and referral. Capable systems include safety, prevention and detection, continuity and integration, timely action and population health management. Positive user experience demands respect for patients, which includes dignity, privacy, non-discrimination, autonomy and clear communication. In addition, there is a need for user focus to have a choice of providers, short wait times, affordability and ease of use. In this study, we consider insured patients (NHIMA members and beneficiaries) and uninsured patients as ‘users’. We examine how the health insurance is designed to improve the experiences of its beneficiaries and if there are spillover effects or unintended consequences of its design and implementation on the general population.

### Data collection and analysis

Key-informant interviews were conducted using a semi-structured interview guide. The guide was designed using constructs from strategic purchasing and HQSS frameworks. The interviews were conducted in English by the first author, and they lasted on average an hour.

After the completion of the interviews, we applied the seven-step framework analysis method. This included transcription, familiarization with transcripts, coding, developing a framework, application of framework, charting and data interpretation (Srivastava and Thomson, 2009). Atlas ti.8 was used to assist in coding.

## Results

The results of the study are presenting the context of the health system in Zambia and the design of the three main purchasing functions of its NHI and their implications to influence the quality of care.

### Health system functions

The health system functions in terms of governance of quality, service delivery and financing are essential to the extent to which a purchaser such as the NHIMA can achieve its goal of improving access to quality care (Cashin *et al.*, 2018). However, in Zambia, our analysis of the document review and key-informant interview points to several governance challenges including mismanagement of public resources (Chansa *et al.*, 2018) such as a major scandal in 2020 with the procurement of about USD 17 million worth of defective health kits and medicines. Although there is a council responsible for licencing health facilities and training institutions, stakeholders perceived its power as a regulator with ‘teeth’ as weak. Quality of care has been highly prioritized with the national quality improvement guidelines in 2017 (Zambia Ministry of Health, 2017) and the performance improvement and Quality assurance strategy 2019–21 (Zambia Ministry of Health, 2019), but the wide variety of definitions of quality of care by stakeholders suggest that the engagement with the documents has been limited. One view particularly those at the lower levels of the health system had the assumption that without adequate structural capacity quality can never be guaranteed. Another view placed a high emphasis on quality from users’ perception in terms of waiting times and the availability of health workers and medicines. Interviewees explained that the perception of quality centred on medicines and diagnostics, as those have been the major public concern.

There have also been financial challenges with public health spending declining over the years. The share of general government expenditure on current health expenditure (CHE) was 7.1% in 2016, substantially below the 15% target of Abuja (Zambia Ministry of Health, 2018a). Meanwhile, donors contribute ∼42% to the total CHE of which 70% is earmarked for specific diseases (Zambia Ministry of Health, 2018a). The government’s deficit in health spending has been compounded by a major macroeconomic crisis coupled with the coronavirus disease 2019 pandemic, which has shifted the government’s priorities towards debt repayment (Zambia Ministry of Finance, 2020; Geda, 2021; Paul *et al.*, 2021). The low public health spending has affected the financial resources health facilities receive, with 3 out of the 12 monthly grants disbursed the year before the insurance implementation. Even with the low public health spending, expenditure is not uniform across the health system, with larger proportions dedicated to hospitals compared with primary care (Chansa *et al.*, 2018). Meanwhile, stakeholders perceived payroll contributions to the health insurance were not likely to be sufficient for major improvements in quality of care as the formal sector, which has the majority of the insurance members, is very small (Zambia Statistics Agency, 2019).

The governance and financing challenges have been detrimental to the quality of service provision, particularly at the primary healthcare level (Chansa *et al.*, 2018). Although primary health care is ‘free’, due to the shortage of medicines and supplies, users sometimes pay out of pocket (Chansa *et al.*, 2019). The funding challenges have also affected filling the human resource for health gaps for services such as surgery, obstetrics and anaesthesia (Zambia Ministry of Health, 2018b).

The service delivery challenges are not homogeneous across geographical locations. In rural areas, the main challenge is physical access as hospital services are located in district and provincial centres. The Zambia Flying Doctors were established to aid in transporting patients to higher levels of care, but this service is not fully functional across the country. In urban areas, long waiting times is the major issue, particularly in hospitals partly due to bypassing lower levels of care although there is a referral guideline and bypassing fee policy in some hospitals.

To improve service readiness for the implementation of the health insurance, the NHIMA provided claims advanced payment (CAP) to health facilities to make short-term investments for quality improvement. However, some stakeholders perceived this payment not to be sufficient as the facilities needs were far greater. CAP is based on the monthly grant from the MOF, which uses the needs-based resource allocation formula that has been difficult to fully apply due to the proliferation of new districts (Chansa *et al.*, 2018).

### Healthcare goods and services to purchase

The health insurance requires members to make four consecutive contributions before accessing health services. Services include a range of essential services such as caesarean sections and costly interventions including cancer care and dialysis (National Health Insurance Management Authority, 2020). Stakeholders believed that this arrangement would allow individuals to be able to receive high-cost hospital interventions without having to face financial hardship. According to stakeholders, the initial design of the benefit package in 2019 was informed by the national health strategic plan, by the burden of diseases, and in consultation with relevant stakeholders. The health insurance bill is explicit in the use of generic medicines and the establishment of a drug formulary system to discourage the use of ineffective or costly medications. However, for services and medical interventions, there is no clear guidance on mechanisms and the conditions to make systematic revisions to the benefit package. In the absence of a clear regulatory framework to guide the revision of the benefit package, there has been pressure from influential groups to expand the package to include high-cost services such as treatment abroad.

One of the main approaches by the NHIMA in delivering higher-quality services is improving user experiences. To mitigate some of the service delivery challenges in public hospitals, the NHIMA introduced a new tier into the service structure by requiring facilities to have designated inpatient care and a sufficient supply of medicines for its members. In addition, facilities are encouraged to fast-track NHIMA patients for outpatient services and operate a 24/7-hour member access. Providers described the challenges in implementing these institutional reforms. For instance, a manager in a Level 2 hospital elaborated that the hospital had already outgrown the population it serves, and creating wards specifically for NHIMA patients is difficult. Furthermore, they mentioned that an insufficient workforce makes it demanding to have adequate staff dedicated to NHIMA patients. Some stakeholders felt that the reforms raise equity concerns.

The concept of NHIMA, they wanted everybody to be receiving the quality service. Now because of the challenge, we’ve seen in some institutions now, they are trying to reserve drugs. ‘No this is for NHIMA members and this is for ordinary members’. Ordinary person will come, they will say, ‘There is no Panadol. Go and buy.’ But a NHIMA person will come and then they will give. But that’s not what we are encouraging. We are saying all patients should receive the health services because we need to raise the standard at all our institutions. (Provincial KI)

Those at the facility level argued for the decision to create separate services for NHIMA members. One is the need to show the benefit of the health insurance for members compared with the general population.

So the NHIMA client is a paying client so the money that they are giving us is been deducted through their pay slip. So of course it’s something that is mandatory with the laws of Zambia however, we find that once you are paying for service and you are in queue with everyone else even with some who are not paying … it really puts a damper on the patient experience. By separating the NHIMA patients to sort of like … if I could say fast track them getting their service for which they are paying for, we believe that this will make their experience here at the hospital more enjoyable and more comfortable. (Health facility KI)

### Providers from whom goods and services are purchased

As user fees had already been abolished at the primary care level, the NHIMA had to cover services offered above Level 1 hospitals. However, Level 1 hospitals were included as their exclusion would have restricted access to care in rural areas. There were concerns raised by some stakeholders of the efficiency implications of this new arrangement with the current MOH referral guidelines. There were interpretations that the health insurance being offered at high levels, and insurance members and service providers will exacerbate the bypassing of lower levels of care.

NHIMA is a business. Even when you enter this institution, it’s a business. If a customer is entering your shop, do you chase them? So you won’t chase the customer so even here, that is the concept … maybe even the institution, the management they should have that focus. Because here, a NHIMA client comes, then you say, ‘No you go and start with the clinic’. What are you losing? You are losing resources. (Provincial KI)

Stakeholders mentioned that the inclusion of private providers could improve the integration of the different service providers in the health system and increase the choices of scheme members as the health insurance allows beneficiaries to use private pharmacies in the instance of drug stock outs. Others also perceived the inclusion of the private sector to be a good strategy to decongest public health facilities in urban areas. However, there were three main concerns raised by others about the inclusion of the private sector. One was the implications for inter-facility communication and referral networks for the diverse service providers as the national referral policy pertains only to the public sector.

The second concern is the efficiency implications of using private pharmacies in filling the gaps in the public health sector.

Our pharmacies in the public facilities should have those drugs. There is no reason why we should be encouraging our public facilities to write a prescription to a private pharmacy. It doesn’t make logical sense to me. Because we should encourage the public facility to recoup everything. Remember this guy is using government time, is paid by the government, (Mbau et al.) writes a prescription for a private facility to benefit. (National KI)

Lastly are the equity implications of who benefits from the inclusion of private providers. In 2019, there were 543 registered private health facilities including diagnostic centres, but ∼80% were located in two provinces, and most providers were concentrated within the urban districts of these provinces (Health Professions Council of Zambia, 2019).

One of the main approaches that the NHIMA is using to guarantee quality from all providers is through accreditation. Service providers that have valid licence and are fully compliant with their relevant regulatory bodies are eligible to apply for accreditation. There is an accreditation checklist developed for the various service providers. A review of the quality indicators of the accreditation and inspection tools showed the assessment to be heavily focused on the structural capacity of providers with less emphasis on process or impact indicators. Even with the current accreditation tools, policymakers acknowledged that due to the persisting health system challenges not all accredited health facilities, particularly those in the public sector, met the accreditation standards. This has been the need to balance access and quality as being stringent on the standards would have cut-off beneficiaries in remote areas from having access to the scheme.

The Act also provides the NHIMA the power and authority to remove health facilities that do not comply with its standards and regulation from its list of accredited health facilities. However, some stakeholders were skeptical of NHIMA actually exercising its power over health facilities which do not comply with its regulations due to previous experiences of officials in charge of quality programmes been removed for exercising their authority.

### How to purchase

The health insurance has mixed payment methods. Accredited pharmacies and diagnostic centres are paid by fee-for-service. Level 1 hospitals receive a flat rate payment with different rates for inpatient and outpatient services. At Level 2 and 3 hospitals, the flat rate payment is also used for outpatient services, but the payment for inpatient services are diagnosis-related groups (DRGs) and fee-for-service for high-cost interventions such as dialysis and some cardiac interventions. Level 2 and 3 hospital managers mentioned that funding from the NHIMA has made a difference in supplementing the purchase of commodities such as essential medicines. However, they acknowledged that this increased funding is not adequate to close the gap in providing high-quality services. Providers reflected that even with the resources from the NHIMA, there is a greater need to improve physical infrastructure and procure medical equipment. As the rates for first levels and higher levels are substantially different, Level 1 hospitals deemed this not to be fair as they also provide some of the inpatient services at higher levels. In addition, some stakeholders mentioned the need to shift to the traditional per-capita payment for Level 1 hospitals, whereby providers are paid in advance. Besides the differences in the payment mechanisms, stakeholders also mentioned that there have been reimbursement delays, which can lead to the interruption of service provision.

However, according to the Health Insurance Act, and the memorandum of understanding between the NHIMA and MOH, the NHIMA has 90 days to reimburse health facilities after claims submission. Some stakeholders mentioned that the delays were partly caused by health facilities due to delays in submitting claims and erroneous filing of claims Furthermore, the NHIMA office being based in Lusaka had made it challenging to resolve claim issues promptly for providers farther from the city.

### Governance in purchasing

A critical element in the governance of purchasing is having effective information systems to monitor the quality of care, provider behaviour and process claims. Meanwhile, in Zambia, there are various electronic health systems in public health facilities, which are uncoordinated and have created information silos (Zambia Ministry of Health, 2018b). In addition, there are low levels of computer literacy in health facilities and underdeveloped technological structure. The claim process is manual, and some health facilities mentioned that it was cumbersome and increased the likelihood of billing errors.

Although there are clear roles and responsibilities for the NHIMA and MOH on paper (Zambia Ministry of Health, 2020), there is still a conflict of interest among the purchasing institutions. The Health Insurance Act provides a considerable amount of power to a ‘Minister’ who in 2021 was the Minister of Health. This minister in collaboration with the NHIMA is in charge of activities such as appointing members of the NHIMA supervisory board, prescribing provider payment methods and reporting requirements for accredited health facilities in which the majority are under the Ministry of Health (Government of Zambia, 2018).

As previously mentioned, in the public health sector, the NHIMA is currently relying on the referral policy of the Ministry of Health, which has challenges in enforcement. In addition, there are currently no mechanisms to coordinate service delivery from both the private and public sectors. Furthermore, with the addition of private pharmacies, there are no existing mechanisms for monitoring prescription patterns or adherence to the rational use of medicines.

Strong technical capacity is needed by the NHIMA to be a strategic purchaser, which can influence access to quality. These technical activities include actuarial analysis, information technology, health technology assessment and quality auditing. There is limited technical capacity to carry out such activities.

The Health Insurance Act provides a legal basis for the rights of all beneficiaries to have equitable access to quality health services. In addition, the act states the importance of transparency and accountability of the health insurance to beneficiaries. As most public health facilities are ‘free’, there is now a high expectation of the services from the NHIMA by the public.

To improve clarity about the scheme and accountability for quality services, the NHIMA has created various tools to empower members about their benefits and their rights to high-quality services. A health complaints committee has been established, which is in charge of hearing and determining matters related to accredited healthcare providers and the NHIMA. Individuals not satisfied with the committee’s decision are allowed to take their case all the way to the High Court and must be compensated by the NHIMA if they win. The NHIMA has also established an online platform for grievances against accredited health providers, a 24/7 call centre and an NHIMA agent in some accredited health facilities to respond to enquiries. There are also representatives of various employee associations in the public and private sectors on the NHIMA’s board. The biggest hurdle has been the lack of clarity about the limits of the benefit package and the obscurity in the mandate of the Ministry of Health and the NHIMA.

## Discussion

The results presented here suggest that Zambia’s NHI is designed to make progress towards strategic purchasing of quality care. With the inclusion of private providers, the insurance has the potential to increase the choices of citizens, particularly those in the urban areas, and integrate the public and private health sectors. Furthermore, unlike other LMICs, which started with schemes for specific groups, Zambia committed from the outset of the insurance scheme to include everyone under its health insurance. This is an important feature as the experience of several countries showed that incrementally expanding the population groups included in health insurance schemes is extremely challenging (Kutzin, 2013; Bazyar *et al.*, 2021). However, similar to other low- and upper-middle-income settings, our findings suggest that significant changes are needed in the purchasing arrangements for the health insurance’s ability to influence high-quality care (Chukwuma *et al.*, 2021; Amporfu *et al.*, 2022; Gatome-Munyua *et al.*, 2022).

Our findings show that the health system challenges in regulatory structures, government health spending and effective referral policy have hindered the design and the early phase of its implementation of the NHI as a purchaser to impact a high quality of care. First, the limited regulatory bodies with teeth to enforce high-quality health system inputs coupled with low government health spending have led to the NHIMA not being able to leverage its role to ensure that all of its providers met its defined quality standards for accreditation. The scheme had to balance access to its benefits and quality of care. Second, the low government spending on health has subsequently led to perpetual drug shortages, long waiting times and poor facility infrastructure in health facilities, which have resulted in signals to providers to distinguish services for beneficiaries to improve their care experiences, an operational challenge for providers. Third, weak enforcement of the national referral policy for gatekeeping could exacerbate the unnecessary use of care at higher levels through opportunistic behaviours by providers and insurance beneficiaries.

The design features of the NHI also face certain shortcomings as a strategic purchaser for quality. First, the scheme’s accreditation and monitoring tools on providers’ performance are heavily reliant on structural quality indicators. Although the structural aspect of quality is a challenge in Zambia, evidence has shown that the relationship among these dimensions is not always hierarchal, and weakness in structural quality does not imply processes of care and impacts cannot be monitored (Kruk *et al.*, 2018a; Quentin and Brownwood *et al.*, 2019). Second, during the study period, the claims submission process was manual, which is prone to errors and low uptake of claims data to monitor performance. To monitor the quality of care effectively, a robust information management system is crucial for the timely use of claims data for quality improvements (Weiner *et al.*, 1990; Konrad *et al.*, 2019; Ng *et al.*, 2019). Since the study period, the NHIMA has established an electronic claim processing system, a substantive milestone in providing data for decision-making and learning. Although the system is in its infancy, it offers the opportunity to use claims to monitor the care given across by different providers. Third, the reimbursement timeframe of 90 days stipulated by the Health Insurance Act is too long for providers to adequately maintain quality of care through the procurement of medicines and other essential consumables. Similar reimbursement delays have been reported in India and Ghana, whereby providers subsequently limited services to insurance members (Boyanagari and Boyanagari, 2019; Akweongo *et al.*, 2021).

Zambia’s NHI is implementing a blended provider payment through fee-for-service, DRGs and flat rate payment systems for hospital care. However, the low rates for Level 1 hospitals could undermine the motivation and quality of care from these providers. Since the study period, the NHIMA has increased the rates for Level 1 hospitals, which shows that the NHIMA is learning as a purchaser to use the information to make the necessary changes. It will be vital to assess in the future whether the new rates in Level 1 hospitals and the scheme’s payment methods are creating the right incentives to influence the quality of care by providers. Appropriate referrals and coordination of care among the different levels are still not part of the provider payment mechanism. As Level 1 hospitals are at the bottom of the referral system under the scheme, the NHIMA can incentivize them for appropriate referrals and coordination to higher levels of care.

Based on the findings, we provide recommendations on how the NHI in Zambia can leverage to make a higher influence on the quality of care. First, with the launch of pay-for-performance (P4P) under health insurance, there is potential for greater influence on providers’ behaviour for a higher quality of care. A systematic review of P4P found that process and intermediary outcome indicators are more likely to affect the quality of care (Van Herck *et al.*, 2010). Zambia can leverage its NHI’s P4P for a higher quality of care from its diverse providers through the selection of process and outcome indicators that account for patient safety, appropriate treatment, patient satisfaction ratings and clinical outcomes (Hussein *et al.*, 2021). Second, efforts towards improving the quality of care should also consider equitable access to these high-quality services. As geographical access to the higher-level facilities where the scheme operates is a challenge for a significant proportion of Zambians, investments in referral networks and inter-facility communication will be crucial. In addition, equity concerns raised about the involvement of the private health sector need to be addressed. Since the study period, the accreditation of private providers has continued to mirror the unequal geographical distribution of private providers within the health system. The NHIMA may have to consider including the cost of transportation from remote communities to areas, which have private providers in the instances of a shortage of supplies in the public sector. Third, the actual implementation and enforcement of the accountability actions to improve the quality of care as stipulated by the Health Insurance Act will require the NHIMA and its leadership to be insulated from political interferences. During the study period, the NHIMA was under the MOH, but a change in government in August 2021 moved the authority to MLSS. This shift may be a path towards autonomy from the MOH, whose leadership had the sole responsibility to appoint the NHIMA’s supervisory board and lead the development of statutory instruments. To improve accountability for all Zambians, representation on the board from non-governmental organizations, which represent vulnerable groups such as those unemployed and disabled, should be considered.

This study had some limitations. As with any analysis pertaining to health system reforms, its results are highly time-bound to the study period. As stated previously, there have been several changes within the scheme since the study period including the payment mechanism and claims management system. Further research should assess the effects of these new changes. In addition, the study heavily focused on stakeholders in the public sector, as public health facilities were the major providers of the scheme at the time. There is also a likelihood of selection bias from the key-informant interviews as three stakeholders either declined or could not be reached. As health insurance was relatively new, it is possible that stakeholders with different views about insurance were less likely to participate in the study. However, we corroborated the interviews with the document review to reduce selection bias.

## Conclusion

We drew upon conceptual frameworks on strategic purchasing and quality of care to examine how the design of Zambia’s NHI scheme may affect access to quality care. While still in its infancy, the design of the purchasing arrangements of health insurance appears to be in the right direction despite some shortcomings. More progress towards strategic purchasing for quality of care is likely possible with government contribution to the scheme for vulnerable groups, increased investments in primary health care and a larger and better-qualified health workforce, good governance for quality and an effective referral system within the entire health system. Health insurance can also positively influence the quality of care through a balance of structural, process and outcome indicators to monitor providers and the use of the claims data across its mix of providers.

## Data Availability

Summaries of the interview transcripts are available from the corresponding author upon reasonable request.

## Reflexivity statement

The authors include one female and three males and span multiple levels of seniority. While three of the authors are health economists specializing in health financing reforms related to health insurance in sub-Saharan Africa, the third has expertise in conducting qualitative fieldwork in the Global South. Three of the authors have extensive expertise in conducting health systems research in Zambia.

## References

[R1] Akweongo P, Chatio ST, Owusu R et al. 2021. How does it affect service delivery under the National Health Insurance Scheme in Ghana? Health providers and insurance managers perspective on submission and reimbursement of claims. *PLoS One* 16: e0247397. 10.1371/journal.pone.0247397 PMC7924798 33651816

[R2] Amporfu E, Agyei-Baffour P, Edusei A, Novignon J, Arthur E. 2022. Strategic health purchasing progress mapping: a spotlight on Ghana’s National Health Insurance Scheme. *Health Systems & Reform* 8: e2058337. 10.1080/23288604.2022.2058337 35695801

[R3] Bazyar M, Yazdi-Feyzabadi V, Rashidian A, Behzadi A. 2021. The experiences of merging health insurance funds in South Korea, Turkey, Thailand, and Indonesia: a cross-country comparative study. *International Journal for Equity in Health* 20: 66. 10.1186/s12939-021-01382-w PMC7913450 33637090

[R4] Boyanagari M, Boyanagari VK. 2019. Perceptions and experiences of healthcare providers and beneficiaries on the National Health Insurance Scheme of Rashtriya Swasthya Bima Yojana (RSBY) in a Taluk of South Indian State of Karnataka. *Clinical Epidemiology and Global Health* 7: 136–9.

[R5] Cashin C, Nakhimovsky S, Laird K et al. 2018. Strategic Health Purchasing Progress: A Framework for Policymakers and Practitioners. Bethesda, MD: Health Finance & Governance Project, Abt Associates.

[R6] Chansa C, Matsebula T, Piatti M et al. 2019. *Zambia Health Sector Public Expenditure Tracking and Quantitative Service Delivery Survey*. Washington, DC: World Bank.

[R7] Chansa C, Workie NW, Piatti M, Mastsebula T, Yoo KJ. 2018. Zambia Health Sector Pubic Expenditure Review. Washington, DC: World Bank.

[R8] Chitah BM, Chansa C, Kaonga O, Workie NW. 2018. Myriad of health care financing reforms in Zambia: have the poor benefited? *Health Systems & Reform* 4: 313–23. 30395765 10.1080/23288604.2018.1510286

[R9] Chukwuma A, Lylozian H, Gong E. 2021. Challenges and opportunities for purchasing high-quality health care: lessons from Armenia. *Health Systems & Reform* 7: e1898186. 10.1080/23288604.2021.1898186 33914676

[R10] Duran D, Bauhoff S, Berman P et al. 2020. The role of health system context in the design and implementation of performance-based financing: evidence from Cote d’Ivoire. *BMJ Global Health* 5: e002934. 10.1136/bmjgh-2020-002934 PMC7528372 32999053

[R11] Gatome-Munyua A, Sieleunou I, Barasa E et al. 2022. Applying the strategic health purchasing progress tracking framework: lessons from nine African countries. *Health Systems & Reform* 8: e2051796. 10.1080/23288604.2022.2051796 PMC7613345 35446229

[R12] Geda A . 2021. The Economic and Social Impact of COVID-19 in Zambia. Geneva, Switzerland: United Nations Conference on Trade and Development.

[R13] Government of Zambia . 2018. The National Health Insurance Act [No. 2 of 2018]. Lusaka.

[R14] Government of Zambia. 2019. Statutory Instrument No. 63 of 2019. https://www.nhima.co.zm/download/document/813df761802019102159cc9cc7.pdf, accessed 23 August 2022.

[R15] Health Professions Council of Zambia. 2019. List of private health facilities as 1st December 2019. https://www.hpcz.org.zm/wp-content/uploads/2019/12/Active-Private-Health-Facilities-as-at-1st-December-2019.pdf, accesssed 8 July 2022.

[R16] Hussein M, Pavlova M, Ghalwash M, Groot W. 2021. The impact of hospital accreditation on the quality of healthcare: a systematic literature review. *BMC Health Services Research* 21: 1057. 10.1186/s12913-021-07097-6 PMC8493726 34610823

[R17] International Monetary Fund . 2020. World Economic Outlook Database. International Monetary Fund. https://www.imf.org/en/Publications/WEO/weo-database/2019/October, accessed 5 June 2022.

[R18] Konrad R, Zhang W, Bjarndóttir M, Proaño R. 2019. Key considerations when using health insurance claims data in advanced data analyses: an experience report. *Health Systems (Basingstoke, England)* 9: 317–25. 33354323 10.1080/20476965.2019.1581433 PMC7738306

[R19] Kruk ME, Gage AD, Arsenault C et al. 2018a. High-quality health systems in the Sustainable Development Goals era: time for a revolution. *The Lancet Global Health* 6: e1196–252. 30196093 10.1016/S2214-109X(18)30386-3 PMC7734391

[R20] Kruk ME, Gage AD, Joseph NT et al. 2018b. Mortality due to low-quality health systems in the universal health coverage era: a systematic analysis of amenable deaths in 137 countries. *The Lancet* 392: 2203–12. 10.1016/S0140-6736(18)31668-4 PMC6238021 30195398

[R21] Kutzin J . 2013. Health financing for universal coverage and health system performance: concepts and implications for policy. *Bulletin of the World Health Organization* 91: 602–11. 23940408 10.2471/BLT.12.113985 PMC3738310

[R22] Lagomarsino G, Garabrant A, Adyas A, Muga R, Otoo N. 2012. Moving towards universal health coverage: health insurance reforms in nine developing countries in Africa and Asia. *The Lancet* 380: 933–43. 10.1016/S0140-6736(12)61147-7 22959390

[R23] Mathauer I, Dale E, Jowett M, Kutzin J. 2019. Purchasing Health Services for Universal Health Coverage: How to Make It More Strategic? Geneva: World Health Organization.

[R24] Mbau R, Barasa E, Munge K et al. 2018. A critical analysis of health care purchasing arrangements in Kenya: a case study of the county departments of health. *The International Journal of Health Planning and Management* 33: 1159–77. 30074642 10.1002/hpm.2604 PMC6492197

[R25] National Health Insurance Management Authority . 2020. Release of benefit package for the National Health Insurance Scheme. Lusaka, Zambia: National Health Insurance Management Authority.

[R26] National Health Insurance Management Authority . 2022. Update on the Performance & Status of the National Health Insurance Scheme. Lusaka: NHIMA.

[R27] Ng M, Fullman N, Dieleman JL et al. 2014. Effective coverage: a metric for monitoring universal health coverage. *PLoS Medicine* 11: e1001730. 10.1371/journal.pmed.1001730 PMC4171091 25243780

[R28] Ng JYS, Ramadani RV, Hendrawan D, Duc DT, Kiet PHT. 2019. National Health Insurance Databases in Indonesia, Vietnam and the Philippines. *PharmacoEconomics - Open* 3: 517–26. 30859490 10.1007/s41669-019-0127-2 PMC6861401

[R29] Paul VB, Finn A, Chaudhary S, Mayer Gukovas R, Sundaram R. 2021. COVID-19, Poverty, and Social Safety Net Response in Zambia. *Policy Research Working Paper; No. 9571*. Washington, DC: World Bank.

[R30] Quentin W, Partanen V Brownwood I et al. 2019. Measuring healthcare quality. In: Busse R, Klazinga N and Panteli D et al. (eds). *Improving Healthcare Quality in Europe: Characteristics, Effectiveness and Implementation of Different Strategies*. Copenhagen, Denmark: European Observatory on Health Systems and Policies, 407–17. 31721544

[R32] Srivastava A, Thomson SB. 2009. Framework analysis: a qualitative methodology for applied policy research. *Journal of Administration and Governance* 4: 72–9.

[R33] Van Herck P, de Smedt D, Annemans L et al. 2010. Systematic review: effects, design choices, and context of pay-for-performance in health care. *BMC Health Services Research* 10: 247. 10.1186/1472-6963-10-247 PMC2936378 20731816

[R34] Weiner JP, Powe NR, Steinwachs DM, Dent G. 1990. Applying insurance claims data to assess quality of care: a compilation of potential indicators. *QRB - Quality Review Bulletin* 16: 424–38. 2101446 10.1016/s0097-5990(16)30404-3

[R35] The World Bank . 2020. World Development Indicators. World Bank Group. https://databank.worldbank.org/source/world-development-indicators, accessed 7 June 2022.

[R36] Zambia Ministry of Finance . 2020. Economic Recovery Programme 2020–2023. Lusaka, Zambia: Ministry of Finance.

[R37] Zambia Ministry of Health. 2017. National Quality Improvement Guidelines, Ministry of Health.

[R39] Zambia Ministry of Health. 2018a. Zambia National Health Accounts 2013–2016. Lusaka, Zambia: Ministry of Health.

[R31] Zambia Ministry of Health . 2018b. Zambia National Health Strategic Plan 2017–2021. Lusaka, Zambia: Ministry of Health.

[R38] Zambia Ministry of Health. 2019. Performance and Quality Strategy 2019–2021. Lusaka, Zambia: Ministry of Health.

[R40] Zambia Ministry of Health . 2020. Memorandum of understanding between National Health Insurance Management Authority and Ministry of Health. Lusaka, Zambia: Zambia Ministry of Health.

[R41] Zambia Statistics Agency 2019. Labour Force Survey Report 2019 First Quarter. *Report*. Lusaka, Zambia: Zambia Statistics Agency.

